# Supplementary material for: Predictive Model for the Assessment of Preoperative Frailty Risk in the Elderly
Source: J Clin Med. 2021 Oct 8;10(19):4612. doi: 10.3390/jcm10194612 (PMC8509404; doi:10.3390/jcm10194612)
Supplement: Supplementary file 1 [file jcm-10-04612-s001.zip › jcm-1379692-supplementary.pdf]

Figure S1. Distribution of emergency operations by surgical departments

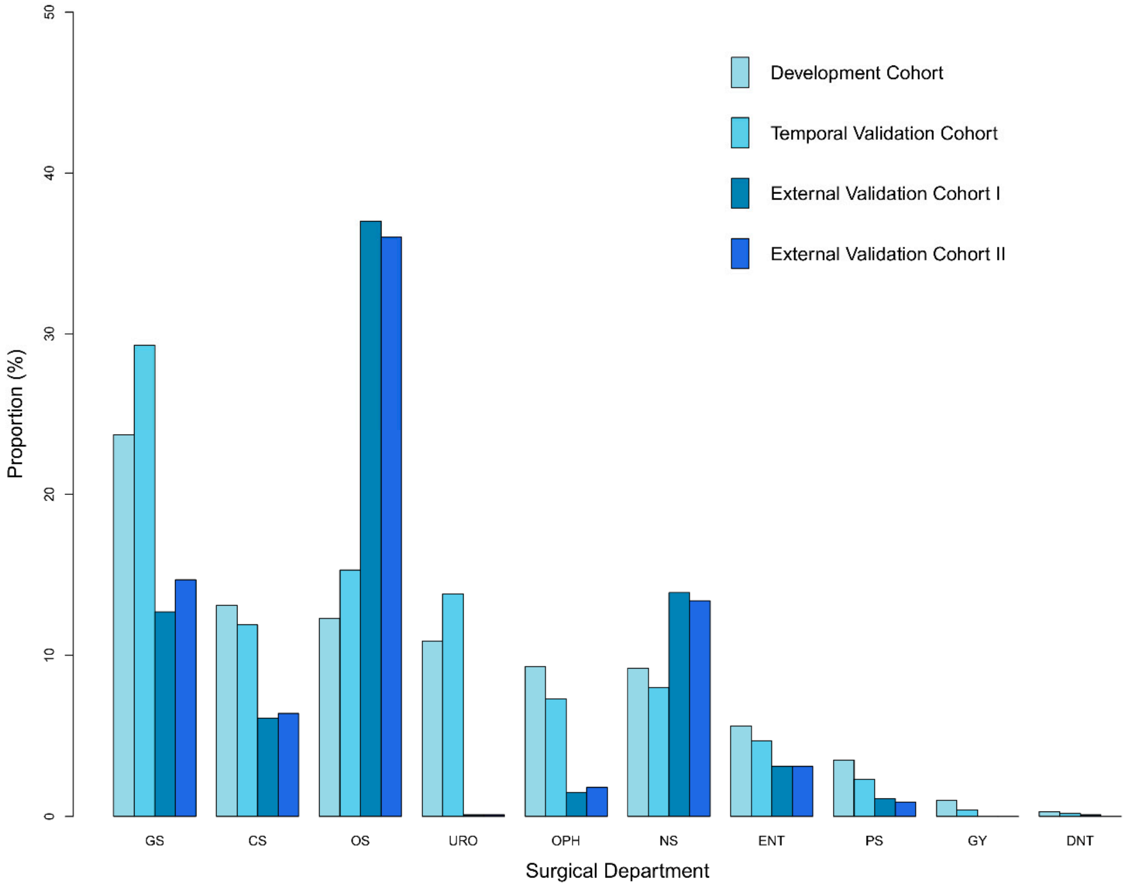

GS, General Surgery; CS, Cardiothoracic Surgery; OS, Orthopedic surgery; URO, Urologic surgery; OPH, ophthalmic surgery; NS, Neuro-Surgery; ENT, Ear, Nose and Throat surgery; PS, Plastic Surgery; GY, Gynecologic surgery; DNT, Dental Surgery.

Figure S2. Distribution of Operation Frailty Risk Score by surgical departments

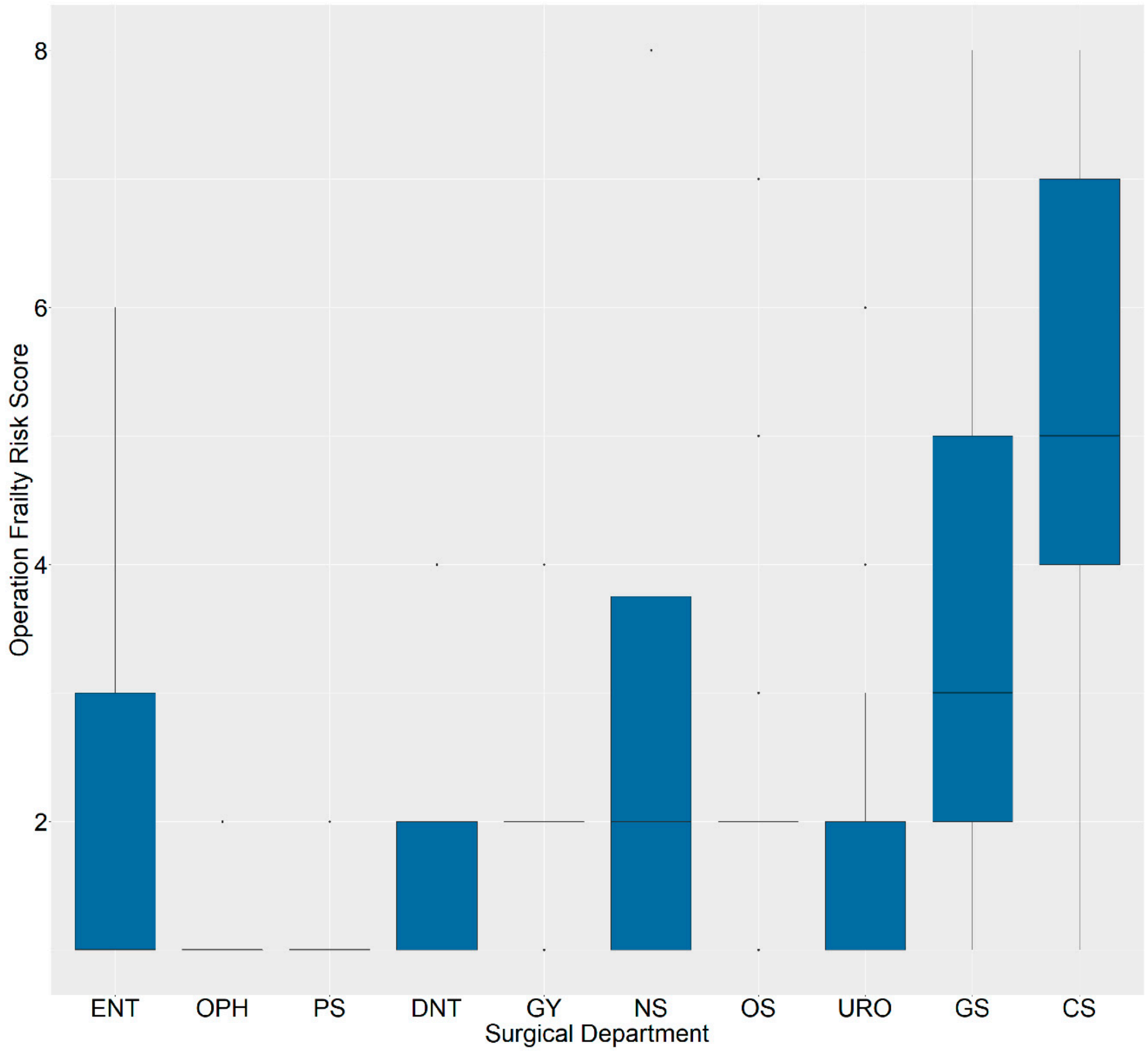

GS, General Surgery; CS, Cardiothoracic Surgery; OS, Orthopedic surgery; URO, Urologic surgery; OPH, ophthalmic surgery; NS, Neuro-Surgery; ENT, Ear, Nose and Throat surgery; PS, Plastic Surgery; GY, Gynecologic surgery; DNT, Dental Surgery.

Table S1. Operation Group classified by clinical experts

OG	Surgical depart	Operation code	Name of operation
1	CS	O1318	Endoscopic Cryotherapy[Tracheal, Bronchial, Lung Tumor]
1	DNT	U4430	Alveoloplasty
1	DNT	U4690	Reduction of Luxated Teeth
1	DNT	U4457	Intraoral Antiphlogosis-Osteitis of Jaw, Osteomyelitis of Jaw etc.
1	ENT	O0951	Single Nasal Polypectomy
1	ENT	S5572	Foreign Body Removal of Ear Canal Or Removal of Impacted Cerumen-Extremely Complex
1	ENT	S5600	Myringotomy
1	ENT	S5620	Ventilation Tube Insertion
1	ENT	O1001	Submucosal Resection or Septoplasty (Cartilage)
1	ENT	O1002	Submucosal Resection or Septoplasty (Bone)
1	ENT	O1004	Incision of Nasal Septal Hematoma or Abscess
1	ENT	O1010	Inferior Turbinectomy
1	ENT	O1011	Inferior Turbinectomy
1	ENT	O1015	Turbinate Outfracture
1	ENT	O1050	Intranasal Antrostomy
1	ENT	O1051	Intranasal Antrostomy
1	ENT	O1092	Operation of Frontal Sinus (Osteoplasty Following Brow Incision)
1	ENT	O1101	Intranasal Ethmoidectomy
1	ENT	O1110	Extranasal Ethmoidectomy
1	ENT	O1120	Intranasal Sphenoidectomy
1	ENT	O1121	Intranasal Sphenoidectomy
1	ENT	O1131	Radical Operation of Pansinusitis
1	ENT	O1141	Radical Operation of Maxillary And Ethmoid Sinus
1	ENT	O1152	Radical Operation of Maxillary, Ethmoid And Sphenoid Sinus
1	ENT	O1161	Radical Operation of Frontal And Ethmoid Sinus
1	ENT	O1176	Radical Operation of Frontal, Ethmoid And Maxillary Sinus
1	ENT	O1215	Removal of Epiglottic Cyst
1	ENT	O1231	Removal of Vocal Nodule or Polyp
1	ENT	O1264	Operation of Vocal Cord Paralysis, Foreign Material Injection (Unilateral)
1	ENT	OA273	Operation of Laryngeal Stenosis, Laser Operation
1	ENT	Q2300	Tonsillectomy
1	GS	M6700	Removal of Biliary Residual Stone
1	GS	M6830	Percutaneous Enterostomy
1	GS	M6850	Cyst Aspiration
1	GS	Q7670	Colonoscopic Removal Of Foreign Body
1	GS	M6721	Fluoroscopic Dilatation of Upper Gastrointestinal Tract Stenosis with Balloon Catheter
1	GS	M6722	Fluoroscopic Dilatation of Upper Gastrointestinal Tract Stenosis with Stent
1	GS	Q7692	Colonoscopic Dilatation Of Colonic Stenosis-Stent Insertion
1	GS	Q7771	Dilatation Of Bile Duct-Simple

1	GS	Q7773	Removal Of Bile Duct Stone
1	GS	Q7774	Removal Of Bile Duct Stone
1	GS	Q7701	Colonoscopic Operation Of Colonic Tumor-Polypectomy
1	GS	Q7702	Colonoscopic Operation Of Colonic Tumor
1	GS	N0900	Excision of Ganglion
1	GS	P2121	Operation For Axillary Lymph Node-Excision
1	GS	P2122	Operation For Axillary Lymph Node-Dissection
1	GS	P2124	Axillary Sentinel Lymph Node-Excision
1	GS	P2141	Operation For Inguinal Lymph Node (Excision)
1	GS	P2142	Operation For Inguinal Lymph Node (Dissection)
1	GS	Q3013	Operation of Hemorrhoids-Hemorrhoidectomy
1	GS	Q7420	Oddi's Sphincterotomy And Sphincteroplasty
1	GS	Q2936	Perineal Operation-Others
1	GS	Q2977	Seton Apply
1	GS	O0266	Extensive Resection of Varicose Vein-Total Stripping of Saphenous Vein, Stab Abulsion of Varices
1	GY	R4521	Dilatation And Curettage
1	GY	R4165	Pelviscopic Fulguration
1	GY	R4240	Polypectomy of Cervical Mucosa
1	GY	R4262	Conization of Cervix-Loop Electrosurgical Excision
1	NS	N0471	Percutaneous Vertebroplasty[Including Discography]
1	NS	N0472	Percutaneous Vertebroplasty[Including Discography], From 2nd Site
1	NS	N0473	Percutaneous Balloon Kyphoplasty[Including Discography]
1	NS	N0474	Percutaneous Balloon Kyphoplasty[Including Discography]
1	NS	N1491	Diskectomy (Invasive)-Cervical Spine
1	NS	N1492	Diskectomy (Invasive)-Thoracic Spine
1	NS	N1493	Diskectomy (Invasive)-Lumbar Spine
1	NS	N2472	Removal of Implant For Internal Fixation of Spine[Posterior]
1	NS	S4595	Neuroplasty-Major Peripheral Plexus
1	NS	S4596	Neuroplasty-Hand And Foot
1	NS	S4825	Radiofrequency Ablation of Spine, Percutaneous-Simple
1	NS	S4843	Implantation of Intrathecal Drug Infusion Pump
1	NS	SY622	Implantation, Change Or Removal of Spinal Neurostimulator Electrodes
1	NS	SY637	Lead Implantation of Spinal Cord Stimulator And Trial Stimulation
1	NS	SY639	Removal of Spinal Cord Stimulator Lead
1	NS	S4851	Navigational Procedure for Surgery -> group 1
1	OPH	S4880	Evisceration
1	OPH	S4891	Removal of Intraocular Foreign Body-With Magnet
1	OPH	S4895	Removal of Intraorbital Foreign Body
1	OPH	S4911	Implantation of Ocular Implant
1	OPH	S4921	Surgery of Corneal Ulcer-Diathermy
1	OPH	S4923	Surgery of Corneal Ulcer-Conjunctival Flap
1	OPH	S4941	Conjunctival Suture
1	OPH	S4950	Excision of Conjunctival Mass
1	OPH	S4960	Curettage of Conjunctival Concretion

1	OPH	S4990	Removal of Scleral Foreign Body
1	OPH	S5000	Scleral Suture
1	OPH	S5011	Scleral Transplantation
1	OPH	S5021	Synechiolysis
1	OPH	S5030	Optical Iridectomy
1	OPH	S5033	Surgery for Glaucoma-Trabeculectomy
1	OPH	S5043	Surgery for Glaucoma-Trabeculectomy
1	OPH	S5044	Surgery for Glaucoma-Photocoagulation for Iris, Ciliary Body
1	OPH	S5049	Glaucoma Implant Surgery
1	OPH	S5050	Vitreous Aspiration
1	OPH	S5091	Anterior Chamber Irrigation
1	OPH	S5111	Surgery for Cataract Or Lens-Extracapsular Or Intracapsular Extraction
1	OPH	S5112	Surgery for After Cataract
1	OPH	S5116	Intraocular Lens Implantation-Secondary
1	OPH	S5117	Intraocular Lens Implantation-Primary
1	OPH	S5118	Intraocular Lens Exchange
1	OPH	S5119	Surgery for Cataract Or Lens-Phacoemulsification
1	OPH	S5121	Vitrectomy-Total
1	OPH	S5122	Vitrectomy-Partial
1	OPH	S5130	Retinal Detachment Surgery
1	OPH	S5140	Cryopexy for Retinal Tear
1	OPH	S5145	Periretinal Membrane Peeling
1	OPH	S5160	Panretinal Photocoagulation
1	OPH	S5161	Endolaser Photocoagulation
1	OPH	S5176	Strabismus Surgery (Complex)-Multiple Muscle
1	OPH	S5231	Removal of Orbital Tumor-Simple
1	OPH	S5245	Excision of Eyelid Tumor-Benign
1	OPH	S5292	Surgery for Blepharoptosis-Muscle Resection
1	OPH	S5293	Surgery for Blepharoptosis-Others
1	OPH	S5321	Conjunctival Sac Reformation-Partial
1	OPH	S5342	Operation of Pterygium-Others
1	OPH	S5371	Keratoplasty-Superficial
1	OPH	S5372	Keratoplasty-Full Thickness
1	OPH	S5374	Keratoplasty-Endothelial Lamellar
1	OPH	S5380	Primary Closure of Cornea
1	OPH	S5421	Suture of Eyelid Laceration-Simple
1	OPH	S5422	Suture of Eyelid Laceration-Through And Through
1	OPH	S5450	Tarsorrhaphy
1	OPH	S5480	Reconstruction of Lacrimal Canaliculi
1	OPH	S5540	Dacryocystorhinostomy
1	OS	M0031	Removal of Foreign Body In Skin, Subcutaneous Tissue or Muscle with Incision of Fascia
1	OS	M0032	Removal of Foreign Body In Skin, Subcutaneous Tissue or Muscle, Others
1	OS	N0641	Closed Reduction of Fractured Extremity[Pelvis,Femur]
1	OS	N0642	Closed Reduction of Fractured Extremity[Humerus,Tibia]

1	OS	N0643	Closed Reduction of Fractured Extremity[Forearm Bone]
1	OS	N0644	Closed Reduction of Fractured Extremity[Carpal Bone,Tarsal Bone]
1	OS	N0645	Closed Reduction of Fractured Extremity[Metacarpal,Metatarsal,Finger,Toe]
1	OS	N0761	Closed Reduction of Dislocation[Hip]
1	OS	N0762	Closed Reduction of Dislocation[Shoulder]
1	OS	N0763	Closed Reduction of Dislocation[Elbow,Knee]
1	OS	N0764	Closed Reduction of Dislocation[Wrist,Ankle,Finger,Toe]
1	OS	N0780	Brisement Force (Manipulation of Joint)
1	OS	N0912	Simple Tendon Suture
1	OS	N0972	Removal of Implant for Internal Fixation of Extremities[Femur]
1	OS	N0973	Removal of Implant for Internal Fixation of Extremities[Humerus,Scapula]
1	OS	N0974	Removal of Implant for Internal Fixation of Extremities[Radius And Ulnar, Tibia And Fibula]
1	OS	N0977	Removal of Implant for Internal Fixation of Extremities[Radius and Ulnar, Tibia and Fibula]
1	OS	N0991	Closed Pinning[Femur]
1	OS	N0995	Closed Pinning[Clavicle,Patella,Carpal Bone,Tarsal Bone]
1	PS	N0210	Operation For Ingrowing Nail
1	PS	N0215	Guttering For Ingrowing Nail
1	PS	N0141	Excision of Skin Benign Tumor (Simple,Superficial)
1	PS	N0142	Excision of Skin Benign Tumor (Others,Extened To Muscle Layer)
1	PS	N0170	Split Thickness Skin Graft-Others (900㎠ Over)
1	PS	N0173	Split Thickness Skin Graft-Face or Joint (25㎠ Below)
1	PS	N0174	Split Thickness Skin Graft-Face or Joint (25㎠ Over)
1	PS	N0175	Split Thickness Skin Graft-Others (25㎠ Below)
1	PS	N0176	Split Thickness Skin Graft-Others (25㎠～100㎠)
1	PS	N0178	Split Thickness Skin Graft-Others (100㎠～400㎠)
1	PS	N0179	Split Thickness Skin Graft-Others (400㎠～900㎠)
1	PS	N0249	Release of Scar Contracture And Flap Operation
1	PS	N0353	Closed Reduction of Nasal Bone Fracture
1	PS	S0161	Skin Flap-Local-Others
1	PS	S0164	Island Flap-Others
1	PS	S0171	Full Thickness Skin Graft (Face)-25㎠ Below
1	PS	S0173	Full Thickness Skin Graft (Extremities)-25㎠ Below
1	PS	S0175	Full Thickness Skin Graft (Others)-25㎠ Below
1	PS	S0176	Full Thickness Skin Graft (Others)-25㎠ Over
1	PS	SA161	Free Flap-Others
1	PS	SA164	Free Comopsite Tissue Flap-Free Musculocutaneous Flap-Others
1	PS	SA165	Free Omental Flap, Free Jejunal Flap-Others
1	PS	SB161	Skin Flap-Local-Face
1	PS	SB165	Muscle Flap-Face
1	PS	SB166	Musculocutaneous Flap or Myocutaneous Flap-Face

1	PS	SB168	Fasciocutaneous Flap-Face
1	PS	SB174	Cadaveric Skin Graft (Others)-25㎠~100㎠
1	PS	SC161	Free Flap-Face
1	PS	SC163	Free Composite Tissue Flap-Free Tendocutaneous Flap-Face
1	PS	SC164	Free Composite Tissue Flap-Free Musculocutaneous Flap-Face
1	PS	N0153	Mohs Micrographicsurgery
1	URO	R3191	Transurethral Ureteral Dilatation-With Balloon
1	URO	R3211	Percutaneous Ureterolithotomy
1	URO	R3264	Ureteral Stent Indwelling-Operative
1	URO	R3267	Removal of Ureteral Stent-Cystoscopic
1	URO	R3424	Flexible Ureterorenoscopic Stone Removal-Kidney
1	URO	R3426	Flexible Ureterorenoscopic Stone Removal-Ureter (Middle)
1	URO	R3563	Operation For Urinary Incontinence-Foreign Material Or Autologous Fat Injection
1	URO	R3565	Operation For Urinary Incontinence-Transvaginal Approach
1	URO	R3566	Operation of Artificial Urethral Sphincter-Removal of Artificial Urethral Sphincter
1	URO	R3567	Operation of Artificial Urethral Sphincter-Insertion of artificial urethral sphincter
1	URO	R3663	Urethroscopic Surgery-Urethral Stent Indwelling
1	URO	R3665	Urethroscopic Surgery-Urethrotomy
1	URO	R4060	Excision of Batholin'S Gland And Cyst
1	URO	RA166	Urinary Fistulectomy-Between Bladder And Intestine
1	URO	R3416	Percutaneous Pyelostomy
1	URO	R3216	Ureteroscopic Ureterolithotomy-Upper
1	URO	R3218	Ureteroscopic Ureterolithotomy-Lower
1	URO	R3381	Renal Pelvis Instillation-By Ureteral Catheterization
1	URO	R3440	Ureterocutaneostomy
1	URO	R3541	Transurethral Bladder Surgery-Tumor (Simple)
1	GS	Q7680	Colonoscopic Bleeding Control
1	GS	Q7730	Sigmoidoscopic Bleeding Control
1	GS	Q7752	Sigmoidoscopic Operation Of Rectosigmoidal Tumor-Mucosal Resection And Submucosal Resection
1	GS	QX706	Colonoscopic Operation of Colonic Tumor-Submucosal Dissection
1	GS	N7133	Mastectomy-Benign (Partial)
1	GS	N7137	Mastectomy-Malignant (Partial)
1	GS	P4551	Total Thyroidectomy-Unilateral
1	GS	P4553	Subtotal Thyroidectomy-Unilateral
1	GS	Q2755	Operation of Inguinal Hernia (Others)-High Ligation
1	GS	Q2722	Operation of Umbilical Hernia-Others
1	GS	Q2756	Operation of Inguinal Hernia (Others)-High Ligation And Posterior Repair
1	GS	Q2757	Operation of Femoral Hernia
1	GS	Q2871	Operation of Internal Bowel Hernia-Reduction
1	GS	Q2881	Operation of Periproctal Abscess (Superficial)-Incision And Drainage

1	GS	Q2883	Operation of Periproctal Abscess-Deep
1	GS	QA753	Operation of Recurrent Inguinal Hernia (With Resection of Intestine)-High Ligation
1	GS	QA756	Operation of Recurrent Inguinal Hernia (Others)-High Ligation And Posterior Repair
1	NS	S4756	CNS Stereotactic Operation-Biopsy, Aspiration, Excision of Lesion, Hematoma Removal
1	NS	S0471	Implantation of Intracranial Neurostimulator Electrodes
1	NS	S0472	Implantation of Electrical Stimulator
1	NS	S0474	Exchange of Electrical Stimulator
1	NS	S4741	Endoscopic Brain Surgery-For Diagnosis
1	NS	S4722	Repair of CSF Leakage-Intraspinal
1	NS	M6599	Percutaneous Cerebral Angioplasty with Drug
1	OPH	S5191	Incision of Orbital Abscess-Invasive
1	OS	N0233	Removal of Subcutaneous Benign Tumor
1	OS	N0317	Osteotomy-Metacarpal, Metatarsal, Finger, Toe
1	OS	N0606	Closed Pinning[Metacarpal,Metatarsal,Finger,Toe]
1	CS	O1532	Removal of Chest Wall Foreign Body (Others)
1	DNT	U4841	Open Reduction of Mandibular Fracture (Symphysis, Body, Angle of Mandible)
1	DNT	U4940	Arthroplasty of TMJ
1	DNT	UX044	TMJ Arthrocentesis
1	ENT	P2113	Neck Lymphatic Dissection-Unilateral (Modified Radical)
1	ENT	P2114	Neck Lymphatic Dissection-Unilateral (Selective)
1	ENT	P2118	Neck Lymphatic Dissection-Bilateral
1	ENT	P2116	Neck Lymphatic Dissection-Unilateral (Modified Radical)
1	ENT	P2117	Neck Lymphatic Dissection-Unilateral (Selective)
1	ENT	P2119	Neck Lymphatic Dissection-Bilateral
1	URO	R3235	Ureteroenterocutaneostomy-Incontinent
1	URO	R3236	Ureteroenterocutaneostomy-Continent
1	URO	R3600	Bladder Neck Plasty
1	URO	R3977	Holmium Laser Enucleation of The Prostate (HoLEP)
2	OPH	S5070	Intravitreal Injection
2	OS	N0922	Fasciotomy, Simple
2	OS	N0923	Fasciotomy, Complex
2	OS	N0041	Escharectomy[9% Under]-Hand, Foot, Finger or Toe
2	URO	R3515	Transurethral Bladder Surgery-Coagulation of Bladder Lesion
2	URO	R3530	Transurethral Resection of Bladder Neck
2	URO	R3540	Transurethral Bladder Surgery-Removal of Blood Clot In Bladder
2	URO	R3576	Cystostomy-Percutaneous
2	URO	R3321	Nephrostomy-Percutaneous
2	URO	R3192	Percutaneous Ureteral Dilatation
2	CS	Q2333	Esophagostomy
2	CS	N0542	Excision of Others
2	CS	O1485	Resection of Chest Wall Tumor, Others (Benign)
2	CS	Q2352	Repair of Hiatal Hernia-Abdominal Approach

2	CS	O0211	Implantation of Cardioverter Defibrillator-Transvenous-Implantation of Cardioverter Defibrillator
2	CS	O1530	Reconstructive Repair of Pectus Excavatum (Ravitch Procedure), Carinatum
2	CS	O1371	Revision of Tracheostoma, Simple
2	CS	O1372	Revision of Tracheostoma With Local Flap
2	DNT	U4533	Surgery of Osteomyelitis of Mandible or Maxilla-Limited Alveolar Bone
2	DNT	U4534	Surgery of Osteomyelitis of Mandible or Maxilla-One Side Mandible 1/3 Below
2	DNT	U4535	Surgery of Osteomyelitis of Mandible or Maxilla-One Side Mandible 1/3 Over
2	DNT	U4456	Intraoral Antiphlogosis-Abscess of Tongue or Mouth of Floor
2	DNT	U4622	Oroantral Fistula Closure with Pedicled Flap
2	ENT	S5711	Decompression of Facial Nerve-Transauricular Approach
2	ENT	S5750	Petrosectomy
2	ENT	S5800	Cochlear Implant
2	ENT	Q2231	Removal of Parotid Tumor-Benign
2	ENT	Q2291	Excision of Oropharyngeal Benign Tumor
2	GS	Q2613	Gastrostomy (Invasive)-Transient
2	GS	Q2614	Gastrostomy (Invasive)-Permanent
2	GS	Q2640	Enterotomy
2	GS	Q2861	Appendectomy-Simple
2	GS	Q2863	Appendectomy-Removal of Appendical Abscess With Periappendical Abscess Drainage
2	GS	Q2862	Appendectomy-Perforated
2	GS	Q2801	Repair of Enteric Fistula-Loop
2	GS	Q2802	Repair of Enteric Fistula-End
2	GS	Q2803	Repair of Enteric Fistula-Double Barrel
2	GS	Q2804	Repair of Enteric Fistula-Reversal of Hartmann Procedure
2	GS	Q7390	Cholecystostomy, Cholecystotomy
2	GS	O2011	External AV Shunt For Hemodialysis
2	GS	P4571	Adrenalectomy-Unilateral
2	GS	Q2551	Vagotomy (Truncal Vagotomy)-With Gastrojejunostomy or Pyloroplasty
2	GS	Q2645	Polypectomy of Small Bowel or Colon
2	GS	Q2561	Pyloroplasty (Fredet-Ramstedt Pyloromyotomy)
2	GS	Q2562	Pyloroplasty (Others)
2	GS	O2081	Fistula Formation-Autologus Vein For Hemodialysis
2	GS	O2082	Fistula Formation-Artificial Vein For Hemodialysis
2	GS	O2083	Repair of Arterio-Venous Fistula For Hemodialysis
2	GY	R4421	Extirpation of Adnexal Tumor-Benign
2	GY	R4148	Hysterectomy (without Lymphadenectomy)-Abdominal approach-complex
2	GY	R0409	Colporrhaphy-Anterior Colporrhaphy
2	GY	R4070	Extirpation of Vaginal Tumor-Benign
2	GY	R4112	Colpopexy (Surgical)-Vaginal Approach
2	GY	R4157	Pelvic And Para-Aortic Lymphadenectomy
2	GY	R4130	Subtotal Hysterectomy

2	GY	R4143	Hysterectomy (With Lymphadenectomy)-Simple
2	GY	R4202	Vaginal Total Hysterectomy
2	GY	R4203	Vaginal Total Hysterectomy With A And P Repair
2	CS	M6521	Percutaneous Atrial Septostomy-Balloon
2	CS	OZ751	Percutaneous Closure of Interatrial Septal Defect
2	CS	M6511	Percutaneous Left Atrial Appendage Occlusion
2	NS	N1497	Laminectomy, Cervical Spine
2	NS	N1498	Laminectomy, Thoracic Spine
2	NS	N1499	Laminectomy, Lumbar Spine
2	NS	N2491	Cervical Spine Laminoplasty
2	NS	N2492	Cervical Spine Laminoplasty
2	NS	N2497	Laminectomy, Cervical Spine
2	NS	N2498	Laminectomy, Thoracic Spine
2	NS	N2499	Laminectomy, Lumbar Spine
2	NS	N0455	Surgical removal of the ossification of spinal ligament (OLF removal-posterior approach)
2	OPH	S4900	Enucleation
2	OPH	S5220	Enucleation and Tissue Implantation
2	OS	N0685	Arthrotomy For Acute Septic Joint[Elbow,Wrist,Ankle]
2	OS	N0688	Arthrotomy For Acute Septic Joint[Elbow,Wrist,Ankle]
2	OS	N0686	Arthrotomy For Acute Septic Joint[Finger,Toe]
2	OS	N0689	Arthrotomy For Acute Septic Joint[Finger,Toe]
2	OS	N2072	Replacement Arthroplasty-Total[Knee]
2	OS	N2077	Replacement Arthroplasty-Total[Knee]
2	OS	N2712	Replacement Arthroplasty-Hemiarhtroplasty[Knee]
2	OS	N3715	Revision of Total Arthroplasty[Ankle]
2	OS	N0311	Ostectomy
2	OS	N0312	Bone Graft
2	OS	N0604	Open Reduction of Fractured Extremity[Clavicle,Patella,Carpal Bone,Tarsal Bone]
2	OS	N0605	Open Reduction of Fractured Extremity[Metacarpal,Metatarsal,Finger,Toe]
2	OS	N0614	Open Reduction of Fractured Extremity[Clavicle,Patella,Carpal Bone,Tarsal Bone]
2	OS	N0622	Repair of Nonunion or Malunion[Humerus,Forearm,Tibia]
2	OS	N0703	Excision of Joint[Including Synovectomy]-Elbow,Wrist,Ankle
2	OS	N0704	Excision of Joint[Including Synovectomy]-Finger,Toe
2	OS	N0708	Excision of Joint[Including Synovectomy]-Elbow,Wrist,Ankle
2	OS	N0710	Excision of Joint [Including Synovectomy]-Knee
2	OS	N0723	Resection Arthroplasty[Finger,Toe]
2	OS	N0733	Arthrodesis[Elbow,Wrist,Ankle]
2	OS	N0734	Arthrodesis[Finger,Toe]
2	OS	N0911	Subcutaneous Tenotomy
2	OS	N0931	Reconstruction of Tendon And Ligament, Simple
2	OS	N0932	Reconstruction of Tendon And Ligament, Complex
2	OS	N0936	Acromioplasty And Repair of Ruptured Shoulder Rotator Cuff (Primary Repair)

2	OS	N0937	Acromioplasty And Repair of Ruptured Shoulder Rotator Cuff (With Myoplasty And Tendoplasty)
2	OS	N0941	Tenolysis
2	OS	N0981	External Fixation[Pelvis,Femur]
2	OS	N0982	External Fixation[Humerus,Tibia]
2	OS	N0986	External Fixation[Humerus,Tibia]
2	OS	N1584	Vascularized Osteocutaneous Graft
2	OS	N1601	Open Reduction of Fractured Extremity-Forearm (Radius)
2	OS	N1604	Open Reduction of Fractured Extremity-Crus (Tibia)
2	OS	N1611	Open Reduction of Fractured Extremity-Forearm (Radius)
2	OS	N1612	Open Reduction of Fractured Extremity-Forearm (Ulnar)
2	OS	N1616	Open Reduction of Fractured Extremity-Crus (Tibia and Fibula)
2	OS	S0165	Muscle Flap-Others
2	OS	S0166	Musculocutaneous Flap or Myocutaneous Flap-Others
2	OS	S0168	Fasciocutaneous Flap-Others
2	OS	N2075	Replacement Arthroplasty-Total[Ankle]
2	OS	N2078	Replacement Arthroplasty-Total[Elbow]
2	OS	N0565	Disarticulation of Extremities[Elbow, Wrist, Ankle]
2	OS	N0566	Disarticulation of Extremities[Finger, Toe]
2	OS	N0582	Revision of Amputated Stump (Need Osteoplasty)-Finger, Toe
2	OS	N0584	Revision of Amputated Stump (Need Plastic Operation On Soft Tissue)-Finger, Toe
2	OS	N0752	Open Reduction of Dislocation[Shoulder]
2	OS	N0282	Curettage or Excision of Benign Bone Tumor[Humerus, Forearm Bone, Clavicle]
2	OS	N0581	Revision of Amputated Stump (Need Osteoplasty)-Thigh, Lower Leg, Upper Arm, Forearm
2	OS	N0583	Revision of Amputated Stump (Need Plastic Operation On Soft Tissue)-Thigh, Lower Leg, Upper Arm, Forearm
2	OS	N0844	Drainage of Intramuscular Abscess (Others)
2	PS	N0952	Operation of Fractured Lower Jaw (Open Reduction)-Symphysis, Body, Angle of Mandible
2	PS	N0152	Wide Excision And Lymph Node Dissection
2	URO	R3542	Transurethral Bladder Surgery-Tumor (Complex)
2	URO	R3543	Transurethral Bladder Surgery-Tumor (Highly Complex)
2	URO	R3290	Partial Nephrectomy
2	URO	R3950	Prostatectomy
2	URO	R3151	Ureteroneocystostomy-Simple
2	URO	R3154	Ureteroneocystostomy-Using Bladder Flap
2	URO	R3180	End-To-End Ureteroureterostomy
2	URO	R3231	Uretero-Intestino-Vesical Anastomosis
2	URO	R3421	Operative Ureterolithotomy-Upper
2	URO	R3620	Repair of Cystocele
2	URO	R3683	Repair of Urethral Stricture-Transpubic
2	URO	R3720	External Urethral Meatotomy
2	URO	R3755	Urethrectomy-Male
2	URO	R3756	Urethrectomy-Female

2	URO	R3770	Removal of Urethral Caruncle
2	URO	R3896	Operation of Vas Deferens-Vasectomy Or Ligation
2	URO	R3400	Pyeloplasty, Ureteroplasty
2	URO	R3433	Ureterectomy-Removal of Remnant Ureter
2	URO	R3461	Cystotomy-Stone Or Foreign Body
2	URO	R3791	Penectomy-Partial
2	URO	R3792	Penectomy-Total
2	URO	R3851	Orchiectomy-Total
2	URO	R3853	Orchiectomy-Partial
2	URO	R3902	Excision of Scrotum-Partial
2	GY	R4427	Extirpation of Adnexal Tumor (Malignant)-Simple
2	NS	N1466	Arthrodesis of Spine-Lumbar Spine-Anterior Technique
2	NS	N1460	Posterior Lumbar Interbody Fusion
2	NS	N1469	Arthrodesis of Spine-Lumbar Spine-Posterior Technique
2	NS	N2468	Arthrodesis of Spine-Cervical Spine-Posterior Technique[C1-2 Fixation]
2	NS	N0466	Arthrodesis of Spine-Lumbar Spine-Anterior Technique
2	NS	N0469	Arthrodesis of Spine-Lumbar Spine-Posterior Technique
2	NS	N2463	Arthrodesis of Spine-Cervical Spine-Anterior Technique[Others]
2	NS	N2466	Arthrodesis of Spine-Thoracic Spine-Anterior Technique[Others]
2	NS	N0468	Arthrodesis of Spine-Thoracic Spine-Posterior Technique
2	NS	N2469	Arthrodesis of Spine-Cervical Spine-Posterior Technique[Others]
2	NS	N2470	Posterior Lumbar Interbody Fusion
2	OS	N0602	Open Reduction of Fractured Extremity[Humerus,Scapula]
2	OS	N0612	Open Reduction of Fractured Extremity[Humerus,Scapula]
2	OS	N3712	Revision of Total Arthroplasty[Knee]
2	OS	N3722	Revision of Total Arthroplasty[Knee]
2	OS	N4712	Revision of hemiarthroplasty[Knee]
2	OS	N4717	Revision of hemiarthroplasty[Knee]
2	URO	R3975	Transurethral Resection of Prostate
2	CS	Q2341	Suture of Esophagus-Cervical Approach
2	CS	Q2342	Suture of Esophagus-Thoracic Approach
2	DNT	U4861	Partial Mandibulectomy
2	ENT	P2112	Neck Lymphatic Dissection-Unilateral (Radical)
2	ENT	P2115	Neck Lymphatic Dissection-Unilateral (Radical)
2	ENT	Q2201	Removal of Tumor In Oral Cavity-Benign
2	ENT	Q2181	Operation for Malignant Tongue Tumor (Less Than One-Half Tongue)
2	GS	Q7566	Pancreatectomy-Wedge Resection
2	GY	R4423	Extirpation of Adnexal Tumor (Malignant)-Simple
2	NS	S4625	Intracranial Foreign Body Removal
2	NS	M6636	Percutaneous Thrombus Removal-Mechanical thrombectomy (Intracranial vessel)
2	NS	M6637	Percutaneous Thrombus Removal-Mechanical thrombectomy (Extracranial cervical vessel)

2	NS	M6639	Percutaneous Thrombus Removal-Mechanical thrombectomy (Others)
2	NS	S4709	Excision of Extradural Tumor Or Lesion-Lumbar Spine-Without Pedicle And Vertebral Body
2	NS	S6696	Excision of Intradural Tumor Or Lesion-Lumbar Spine
2	OS	N2071	Replacement Arthroplasty-Total[Shoulder]
2	OS	N2076	Replacement Arthroplasty-Total[Shoulder]
2	OS	N3716	Revision of Total Arthroplasty[Shoulder]
2	OS	N0701	Excision of Joint[Including Synovectomy]-Hip
2	OS	N0751	Open Reduction of Dislocation[Hip]
2	PS	N0391	Partial Maxillectomy
2	PS	N0404	Partial Maxillectomy, Malignant Tumor
2	PS	N0421	Partial Mandibulectomy
2	PS	N0436	Resection of Malignant Tumor (Partial Mandibulectomy)
2	GS	P4561	Radical Operation of Malignant Thyroid Tumor
2	GY	R4154	Radical Hysterectomy With Bilateral Pelvic Lymphadenectomy (With Para-Aortic Lymph Node Biopsy)
2	GY	R4155	Radical Hysterectomy With Bilateral Pelvic Lymphadenectomy (Without Para-Aortic Lymph Node Biopsy)
2	GY	R4160	Pelvic Adhesiolysis
2	GY	R4424	Extirpation of Adnexal Tumor (Malignant)-Radical
2	GY	R4428	Extirpation of Adnexal Tumor (Malignant)-Radical
2	NS	N0342	Cranioplasty With Dura Graft
2	NS	N0347	Cranioplasty-Complex
2	URO	R3910	Radical Hydrocelectomy
2	GS	OA633	Angioplasty (End-to-End Anastomosis)-Others
2	GS	OA638	Angioplasty (With Patch Graft)-Others,Artificial Vessel
2	GS	OA639	Angioplasty (With Patch Graft)-Others,Autologous Vessel
2	GS	Q7703	Colonoscopic Operation Of Colonic Tumor-Mucosal Resection And Submucosal Resection
2	OS	N0575	Amputation of Extremities[Finger,Toe]
2	PS	N0151	Wide Excision of Skin Malignant Tumor
2	GS	Q7430	Excision of Ampulla of Vater & Replantation of Bile & Pancreatic Duct
2	OS	N2070	Total Arthroplasty[Hip]
2	OS	N0711	Total Arthroplasty[Hip]
2	OS	N2710	Hemiarthroplasty[Hip]
2	OS	N0715	Hemiarthroplasty[Hip]
2	URO	R3960	Total Prostatoseminal Vesiculectomy
2	OS	N1715	Revision of Hemiarthroplasty[Hip]
2	OS	N1725	Revision of Hemiarthroplasty[Hip]
2	OS	N1711	Revision of Total Arthroplasty[Hip]
2	OS	N1721	Revision of Total Arthroplasty[Hip]
2	OS	N3710	Revision of Total Arthroplasty[Hip]
2	OS	N3720	Revision of Total Arthroplasty[Hip]
2	OS	N4710	Revision of Hemiarthroplasty[Hip]
2	OS	N4720	Revision of Hemiarthroplasty[Hip]
2	OS	N0684	Arthrotomy For Acute Septic Joint[Shoulder,Knee]

2	ENT	Q2232	Removal of Parotid Tumor-Malignant
2	GS	Q2775	Primary Repair of Mesentery
3	GS	M6690	Percutaneous Cholecystostomy
3	GS	M6730	Percutaneous Gastrostomy
3	GS	M6741	Percutaneous Abscess Drainage
3	GS	M6750	Percutaneous Drainage Catheter Exchange
3	GS	Q2612	Gastrostomy (Percutaneous)
3	ENT	O1221	Resection of Laryngeal Benign Tumor-Under Endoscopy-Under Suspension Laryngoscopy
3	GS	M6670	Percutaneous Transhepatic Biliary Drainage
3	GS	M6681	Percutaneous Transhepatic Biliary Drainage With Ballooning Catheter
3	GS	M6682	Percutaneous Transhepatic Biliary Drainage With Stent
3	CS	O1510	Closed Thoracostomy
3	CS	O2005	Transcutaneous Cardiac Pacing
3	CS	O1571	Closure of Sternotomy Separation
3	ENT	P2102	Excision of Cervical Lymph Node-Superficial
3	ENT	P2103	Excision of Cervical Lymph Node-Deep
3	GS	Q2810	Adhesiolysis
3	GS	Q2680	Intestinal Anastomosis
3	GS	Q2753	Operation of Inguinal Hernia (With Resection of Intestine)-High Ligation
3	GS	Q2731	Operation of Incisional Hernia-With Resection of Intestine
3	GS	Q2732	Operation of Incisional Hernia-Others
3	GS	Q2754	Operation of Inguinal Hernia (With Resection of Intestine)-High Ligation And Posterior Repair
3	GS	Q2791	Enterostomy-Tube
3	GS	Q2792	Enterostomy-Loop
3	GS	Q2793	Enterostomy-End
3	GS	Q2794	Enterostomy-Double Barrel
3	GS	Q2796	Enterostomy-Revision of Ileostomy or Colostomy (Revision of loop ileostomy)
3	GS	Q2797	Enterostomy-Revision of Ileostomy or Colostomy (Revision of loop colostomy)
3	GS	Q2798	Enterostomy-Revision of Ileostomy or Colostomy (Revision of End ileostomy or End colostomy)
3	GS	Q7380	Cholecystectomy
3	GS	O2073	Vessel Ligation Others
3	GS	O2074	Vessel Ligation Others
3	OS	N0232	Removal of Malignant Tumor
3	URO	R3271	Nephrectomy-Simple
3	URO	R3571	Cystostomy-Operative
3	URO	R3470	Partial Cystectomy
3	URO	R3920	Incision of Scrotal Abscess
3	CS	O1610	Repair of Diaphragmatic Hernia
3	CS	N0550	Radical Curettage of Thoracic Cold Abscess
3	CS	O1562	Sternum Resection And Reconstruction
3	CS	O1600	Repair of Diaphragm
3	CS	Q2424	Esophageal Diverticulectomy-Thoracic Approach

3	CS	O1520	Open Thoracostomy
3	ENT	S5592	Excision of External Auditory Canal Tumor-Malignant
3	GS	Q2710	Intestinal Plication
3	GS	Q2510	Gastrotomy
3	NS	M1661	Embolization-Cerebral/Aneurysm/Assisted
3	NS	M1662	Embolization-Cerebral/Aneurysm/Others
3	NS	M6601	Percutaneous Intravascular Installation of Metallic Stent-Cerebral
3	NS	M6602	Percutaneous Intravascular Installation of Metallic Stent-Carotid
3	OS	N0574	Amputation of Extremities[Hand,Foot]
3	OS	N0591	Open Reduction of Fracture and Dislocation of Spine or Pelvis-Spine
3	OS	N0601	Open Reduction of Fractured Extremity[Femur]
3	OS	N0611	Open Reduction of Fractured Extremity[Femur]
3	NS	M1665	Embolization-Cerebral/Arteriovenous Malformation/Dural Arteriovenous Fistula/Transvenous
3	NS	M1666	Embolization-Cerebral/Arteriovenous Malformation/Caroticocavernous Fistula
3	NS	M1675	Embolization-Tumor/Spinal
3	OS	N0022	Operation of Osteomyelitis or Bone Abscess[Drilling, Fenestration Etc]-Humerus,Forearm Bone,Clavicle
3	OS	N0023	Operation of Osteomyelitis or Bone Abscess[Drilling, Fenestration, Saucerization Etc]-Others
3	OS	N0025	Operation of Osteomyelitis or Bone Abscess[Drilling, Fenestration Etc]-Humerus,Forearm Bone,Clavicle
3	OS	N0026	Operation of Osteomyelitis or Bone Abscess[Drilling, Fenestration, Saucerization Etc]-Others
3	OS	N0021	Operation of Osteomyelitis or Bone Abscess[Drilling, Fenestration Etc]-Pelvis,Femur,Tibia
3	URO	R3241	Augmentation Enterocystoplasty
3	URO	R3482	Total Cystectomy-Others
3	URO	R3273	Nephrectomy-Radical
3	ENT	Q2182	Operation for Malignant Tongue Tumor-Hemiglossectomy
3	ENT	Q2203	Removal of Tumor In Oral Cavity-Malignant
3	CS	O1401	Wedge Resection of Lung, Single
3	GS	O0161	Vascular Bypass Operation (Femoral-Femoral, Clavicle-Clavicle Or Axilla-Axilla),Autologous Vessel
3	GS	O0162	Vascular Bypass Operation (Femoral-Femoral, Clavicle-Clavicle Or Axilla-Axilla),Artificial Vessel
3	GS	O0163	Vascular Bypass Operation (Femoral-Popliteal[Above Knee Joint]),Autologous Vessel
3	GS	O0164	Vascular Bypass Operation (Femoral-Popliteal[Knee Joint Upper]),Artificial Vessel
3	GS	O0166	Vascular Bypass Operation (Femoral-Popliteal[Below Knee Joint]),Artificial Vessel
3	GS	Q2490	Incision And Drainage of Subphrenic Abscess
3	GS	P2094	Splenorrhaphy
3	CS	M6620	Percutaneous Intravascular Installation of Metallic Stent-Percutaneous Intravascular Atherectomy
3	CS	M6597	Percutaneous Transluminal Angioplasty-Others
3	CS	M6613	Percutaneous Intravascular Installation of Stent Graft-Others

3	NS	S4705	Excision of Extradural Tumor Or Lesion-Cervical Spine-Without Pedicle And Vertebral Body
3	NS	S6692	Excision of Intradural Tumor Or Lesion-Cervical Spine
3	NS	S4706	Excision of Extradural Tumor Or Lesion-Thoracic Spine-Involving Pedicle And/Or Vertebral Body
3	OS	N0680	Arthrotomy For Acute Septic Joint[Hip]
3	OS	N0681	Arthrotomy For Acute Septic Joint[Hip]
4	URO	R3432	Ureterectomy-Total Nephroureterectomy
4	GS	Q2691	Operation for Intestinal Obstruction-Including Resection of Intestine
4	GS	Q2692	Operation for Intestinal Obstruction-Entero-Enterostomy
4	GS	Q2693	Operation for Intestinal Obstruction-Adhesiolysis
4	GS	Q7310	Choledochotomy And Choledocholithotomy
4	CS	M6632	Percutaneous Thrombus Removal-Thrombolysis-Others
4	URO	R3251	Intestinal Substitute of Bladder
4	URO	R3272	Donor Nephrectomy
4	URO	R3550	Repair of Bladder Rupture
4	CS	O1605	Excision of Diaphragmatic Tumor And Reconstruction
4	CS	O1645	Vascular Bypass Operation (Artery-Others),Autologous Vessel
4	CS	O1646	Vascular Bypass Operation (Artery-Others),Artificial Vessel
4	CS	O1581	Mediastinostomy, Collor's
4	CS	O2035	Resection of Aneurysm-Others
4	CS	O2037	Resection of Aneurysm-Iliac Artery (Bilateral)
4	CS	O2038	Resection of Aneurysm-Iliac Artery (Unilateral)
4	CS	O1486	Resection of Chest Wall Tumor, Others (Malignant)
4	CS	O1484	Resection of Chest Wall Tumor With Reconstruction of Chest Wall (Malignant)
4	DNT	U4465	Extraoral Antiphlogosis-Deep Layer
4	DNT	U4812	Total Maxillectomy, Malignant Tumor
4	ENT	O1251	Total Laryngectomy And Hypopharyngectomy
4	ENT	O1252	Total Laryngectomy And Partial Hypopharyngectomy
4	ENT	Q2251	Incision of Retropharyngeal Abscess-Intraoral
4	ENT	Q2252	Incision of Retropharyngeal Abscess-Transcervical
4	ENT	O0962	Extirpation of Malignant Tumor of Nasal or Paranasal Sinuses (Radical Maxillectomy)
4	GS	Q2762	Excision of Mesenteric Tumor-Others
4	GS	Q2502	Excision of Retroperitoneal Tumor-Malignant or Pheochromocytoma
4	GS	P2091	Splenectomy-Total
4	GS	Q7221	Hepatectomy-Wedge Resection
4	GS	O2064	Transluminal Atherectomy-Abdominal Artery or Iliac Artery
4	GS	O2067	Transluminal Atherectomy-Abdominal Artery or Iliac Artery
4	GS	Q0257	Subtotal Gastrectomy (Wedge Resection)
4	GS	O2072	Vessel Ligation Following Laparotomy
4	GS	Q7563	Subtotal Pancreatectomy
4	GS	Q7565	Distal Pancreatectomy
4	GS	OA632	Angioplasty (End-to-End Anastomosis)-By Laparotomy

4	GS	OA636	Angioplasty (With Patch Graft)-By Laparotomy,Artificial Vessel
4	GS	OA637	Angioplasty (With Patch Graft)-By Laparotomy,Autologous Vessel
4	GS	Q2673	Colectomy-Segmental Resection
4	GS	QA673	Colectomy-Segmental Resection
4	GS	QA679	Colectomy With Proximal Colostomy And Distal Stump
4	GS	QA921	Rectal And Sigmoid Resection-Anterior Resection
4	GS	QA922	Rectal And Sigmoid Resection-Low Anterior Resection
4	GS	QA923	Rectal And Sigmoid Resection[A-P Resection (Mile's Operation) or A-S Resection]
4	GS	Q2892	Resection of Rectal Tumor-Abdominal Approach
4	GS	Q2921	Rectal And Sigmoid Resection-Anterior Resection
4	GS	Q2922	Rectal And Sigmoid Resection-Low Anterior Resection
4	GS	Q2923	Rectal And Sigmoid Resection[A-P Resection (Mile's Operation) or A-S Resection]
4	GS	QA928	Rectal and Sigmoid Resection-Ultra-Low anterior resection
4	GS	Q2481	Peritoneal Lavage
4	GS	Q2540	Simple Closure of Perforated Stomach or Duodenum
4	GY	R4156	Pelvic Exenteration
4	CS	M6605	Percutaneous Intravascular Installation of Metallic Stent-Others
4	GS	O2065	Transluminal Atherectomy-Others
4	GS	O2068	Transluminal Atherectomy-Others
4	NS	S4711	Shunt Operation Or Bypass Operation-Subdural/Subarachnoid-Other
4	NS	S4712	Shunt Operation Or Bypass Operation-Ventriculo-Other
4	NS	S4801	Operation of Skull Base-Anterior Cranial Fossa
4	NS	N0321	Burr Hole or Trephination For Exploration
4	NS	N0322	Burr Hole or Trephination For Drainage And/Or Evacuation of Cyst,Hematoma or Abscess (Sub or Epidural)
4	NS	N0323	Burr Hole or Trephination For Drainage And/Or Evacuation of Cyst,Hematoma or Abscess (Intracerebral)
4	NS	N0324	Burr Hole or Trephination For Others
4	URO	R3481	Total Cystectomy-Radical
4	NS	N0451	Vertebral Corpectomy (Cervical Spine)
4	NS	N0453	Vertebral Corpectomy (Lumbar Spine)
4	NS	M6644	Embolization-Others
4	NS	S4661	Intracerebral Vascular Anastomosis-Direct
4	NS	N0452	Vertebral Corpectomy (Thoracic Spine)
5	GS	Q2440	Diagnostic Exploratory Laparotomy
5	CS	O1502	Irrigation of Empyema Cavity
5	CS	O1981	Resection of Atrial Myxoma
5	CS	O1982	Resection of Cardiac Tumor-Others
5	CS	O1596	Mediasternal Lymph Node Dissection
5	CS	O1597	Mediasternal Lymph Node Dissection
5	CS	O1591	Excision of Mediastinal Benign Tumor
5	CS	O1410	Segmentectomy of Lung
5	CS	O1586	Mediastinostomy By Thoracotomy

5	CS	O1336	Removal of Tracheal or Bronchial Foreign Body By Thoracotomy
5	CS	O2004	Implantation of Internal Pulse Generator By Thoracotomy
5	CS	O1321	Tracheal or Bronchial Repair, Cervical Approach
5	CS	O2006	Operation of Arrhythmia-Supraventricular Arrhythmia
5	CS	M6542	Conventional Radiofrequency Ablation of Atrial fibrillation
5	CS	M6545	Conventional Radiofrequency Ablation of Atrial fibrillation with Septal Puncture
5	CS	O1440	Repair of Lung
5	ENT	O1224	Resection of Laryngeal Malignant Tumor (Cordectomy)
5	ENT	O1225	Partial Laryngectomy (Vertical, Supraglottic)
5	ENT	O1227	Resection of Laryngeal Malignant Tumor (Total Laryngectomy)
5	ENT	Q2292	Operation for Pharyngeal Malignant Tumor (Oropharyngeal)-Simple Resection
5	ENT	Q2293	Operation for Pharyngeal Malignant Tumor (Oropharyngeal)-Composite Resection
5	ENT	Q2294	Operation for Pharyngeal Malignant Tumor-Partial Hypopharyngectomy
5	ENT	O1300	Invasive Tracheostomy
5	ENT	O0226	Transluminal Atherectomy-Carotid Artery (Simple)
5	ENT	O0227	Transluminal Atherectomy-Carotid Artery (Complex)
5	ENT	O2066	Transluminal Atherectomy-Carotid Artery
5	GS	Q7410	Radical Cholecystectomy of GB Cancer
5	GS	Q0251	Subtotal Gastrectomy (Partial)
5	GS	Q0252	Subtotal Gastrectomy (Distal)
5	GS	Q0253	Subtotal Gastrectomy (Distal)
5	GS	Q0258	Subtotal Gastrectomy (Proximal Resection)
5	GS	Q0259	Subtotal Gastrectomy
5	GS	Q2598	Subtotal Gastrectomy (Proximal Resection)
5	GS	Q2594	Subtotal Gastrectomy (Partial)
5	GS	O2045	Inferior Vena Cava Filter Placement
5	GS	Q2671	Right or Left Hemicolectomy
5	GS	Q1261	Colectomy-Subtotal
5	GS	Q1262	Colectomy-Subtotal
5	GS	QA671	Right or Left Hemicolectomy
5	GS	Q7222	Hepatectomy-Segmentectomy
5	GS	Q7225	Hepatectomy-Bisegmentectomy
5	GS	Q2650	Resection of Small Intestine
5	GS	Q2651	Resection of Small Intestine
5	GS	Q7561	Pancreatectomy-Total
5	GS	Q7592	Pancreaticoenterostomy (End-to-End Anastomosis)
5	GS	Q7351	Choledochoduodenostomy
5	GS	Q7352	Choledochojejunostomy[Roux-en-Y]
5	GS	Q2572	Gastrojejunostomy
5	GS	Q2573	Gastrojejunostomy[Roux-En-Y]
5	GS	Q2771	Repair of Bowel And Mesenteric Injury-With Resection of Intestine
5	GS	Q2773	Serosal Repair or Primary Repair of Perforated Intestine

5	GS	Q2601	Esophagojejunostomy
5	CS	M6595	Percutaneous Transluminal Angioplasty-Aortic
5	CS	M6603	Percutaneous Intravascular Installation of Metallic Stent-Aortic
5	CS	M6611	Percutaneous Intravascular Installation of Stent Graft-Aortic
5	CS	M6612	Percutaneous Intravascular Installation of Stent Graft-Aortic And Iliac
5	CS	M6551	Percutaneous Transluminal Coronary Angioplasty-Single Vessel
5	CS	M6561	Percutaneous Transcatheter Placement of Intracoronary Stent-Single Vessel
5	NS	S4636	Craniotomy for Excision of Brain Tumor-Infratentorial-Simple
5	NS	S4641	Cerebral Aneurysm-Simple
5	NS	S4642	Cerebral Aneurysm-Complex
5	OS	N0562	Disarticulation of Extremities[Hip]
5	OS	N0573	Amputation of Extremities[Upper Arm,Forearm,Lower Leg]
6	CS	O1360	Exploratory Thoracotomy
6	GS	Q7342	Radical Resection of Bile Duct-Malignant
6	CS	O1592	Excision of Mediastinal Malignant Tumor
6	CS	O1841	Repair of Arteriovenous Malformation-By Thoracotomy
6	CS	O2071	Vessel Ligation Following Thoracotomy
6	CS	OA631	Angioplasty (End-to-End Anastomosis)-By Thoracotomy
6	CS	OA634	Angioplasty (With Patch Graft)-By Thoracotomy,Artificial Vessel
6	CS	OA635	Angioplasty (With Patch Graft)-By Thoracotomy,Autologous Vessel
6	CS	O1421	Single Lobectomy of Lung
6	CS	O1422	Bilobectomy of Lung
6	CS	O1660	Repair of Cardiac Wound
6	CS	O1480	Pleurodesis
6	CS	O1460	Apicolysis, Pleurolysis
6	CS	O1932	Creation of Pericardial Window-by Thoracotomy
6	CS	O1935	Creation of Pericardial Window-by Microscopy
6	CS	O1931	Pericardiostomy
6	ENT	O2055	Thrombectomy (Artery),Neck
6	GS	Q2925	Total Coloprotectomy (With Ileostomy)
6	GS	Q2533	Total Gastrectomy-Abdominal Approach
6	GS	Q2536	Total Gastrectomy-Abdominal Approach
6	GS	Q7223	Hepatectomy-Lobectomy
6	GS	O0281	Removal of Infected Graft-Others
6	GS	Q7224	Hepatectomy-Trisegmentectomy
6	GS	QA925	Total Coloprotectomy (With Ileostomy)
6	GS	Q2672	Colectomy-Total
6	GS	QA672	Colectomy-Total
6	GS	Q2679	Colectomy With Proximal Colostomy And Distal Stump
6	GS	O2054	Thrombectomy (Artery),Abdomen
6	GS	O0218	Thrombectomy (Deep Vein),Lower Extremity
6	GS	O2058	Thrombectomy (Deep Vein),Abdomen

6	GS	Q7572	Pancreaticoduodenectomy-Pylorus-Preserving Operation
6	GS	Q7571	Pancreaticoduodenoctomy-Whipple'S Operation
6	CS	M6650	Percutaneous Installation of Inferior Vena Cava Filter
6	CS	M6531	Percutaneous Valvuloplasty-Mitral Valve
6	CS	M6532	Percutaneous Valvuloplasty-Aortic Valve
6	CS	M6580	Transcatheter Aortic Valve Implantation-Transapical Approach
6	CS	M6581	Transcatheter Aortic Valve Implantation-Transaortic Approach
6	CS	M6582	Transcatheter Aortic Valve Implantation-Transfemoral, Transsubclavian Approach
6	GS	O2056	Thrombectomy (Artery),Others
6	GS	O2059	Thrombectomy (Deep Vein),Others
6	NS	S4635	Craniotomy for Excision of Brain Tumor-Supratentorial-Complex
6	NS	S4621	Craniotomy for Evacuation of Hematoma-Subdural Or Extradural
6	NS	S4622	Craniotomy for Evacuation of Hematoma-Intracerebral
6	NS	N0331	Craniotomy or Craniectomy For Exploration
6	NS	N0334	Craniotomy or Craniectomy For Excision of Cranial Lesion or Benign Tumor
6	URO	R3280	Renal Transplantation
7	GS	Q7230	Hepatopancreaticoduodenectomy
7	GS	Q2445	Damage Control Laparotomy
7	CS	O0173	Vascular Bypass Operation (Aorto to carotid and subclavian artery)
7	CS	O2034	Resection of Aneurysm-Abdominal Aorta And Iliac Artery
7	CS	O1431	Pneumonectomy
7	CS	Q2402	Curative Operation of Esophageal Malignant Tumor-Thoracic And Abdominal Approach
7	CS	Q2403	Curative Operation of Esophageal Malignant Tumor-Cervical, Thoracic And Abdominal Approach
7	CS	O1521	Pericardiaolysis with Redo-sternotomy
7	CS	Q2362	Esophageal Bypass Reconstruction-Thoracic And Abdominal Approach
7	CS	Q2366	Esophageal Reconstruction After Resection-With Stomach
7	CS	Q2367	Esophageal Reconstruction After Resection-With Jejunum
7	GS	O0224	Resection of Aneurysm-Abdominal Aorta (Infrarenal)
7	OS	N0572	Amputation of Extremities[Thigh]
7	CS	O1644	Vascular Bypass Op (Aorta-Renal,Thoracic,Abdominal Aorta-Femoral,Aorta-Splanchnic),Artificial Vessel
7	CS	OA640	Vascular Bypass Operation (Aorta-Coronary)-Simple (Off Pump CABG)
7	CS	OA641	Vascular Bypass Operation (Aorta-Coronary),Simple
7	CS	OA648	Vascular Bypass Operation (Aorta-Coronary)-Simple (Off Pump CABG)
7	CS	OA649	Vascular Bypass Operation (Aorta-Coronary)-Simple (Off Pump CABG)
7	CS	O1640	Vascular Bypass Operation (Aorta-Coronary)-Simple
7	CS	O1641	Vascular Bypass Operation (Aorta-Coronary),Simple
7	CS	O1648	Vascular Bypass Operation (Aorta-Coronary)-Simple
7	CS	O1649	Vascular Bypass Operation (Aorta-Coronary)-Simple

7	CS	O1781	Valvuloplasty-Tricuspid Valve
7	CS	O1782	Valvuloplasty-Mitral Valve
7	CS	O1783	Valvuloplasty-Aortic Valve
7	CS	O1791	Valve Replacement-Tricuspid Valve
7	CS	O1792	Valve Replacement-Mitral Valve
7	CS	O1793	Valve Replacement-Aortic Valve
7	CS	O1799	Sutureless Aortic Valve Replacement
8	CS	OA647	Vascular Bypass Operation (Aorta-Coronary),Complex
8	CS	O1647	Vascular Bypass Operation (Aorta-Coronary),Complex
8	CS	O1794	Reoperation of Valvuloplasty-Tricuspid Valve
8	CS	O1795	Reoperation of Valvuloplasty-Mitral Valve
8	CS	O1796	Reoperation of Valvuloplasty-Aortic Valve
8	CS	O1823	Left Ventricular Aneurysmectomy
8	CS	O1825	Left Ventricular Outflow Track Augmentation
8	CS	O1950	Pulmonary Artery Embolectomy
8	GS	Q8040	Liver Transplantation from Cadaver Donor-Total
8	GS	O0223	Resection of Aneurysm-Abdominal Aorta (Suprarenal[Juxtarenal])
8	CS	O1722	Operation of Ventricular Septal Defect-Post Infarction
8	CS	O2031	Resection of Aneurysm-Ascending Aorta
8	CS	O2032	Resection of Aneurysm-Aortic Arch
8	CS	O2033	Resection of Aneurysm-Descending Thoracic Aorta
8	NS	N0333	Craniotomy or Craniectomy For Decompression

OG, Operation Group; GS, General Surgery; CS, Cardiothoracic Surgery; OS, Orthopedic surgery; URO, Urology; OPH, ophthalmic surgery; NS, Neuro-Surgery; ENT, Ear, Nose and Throat surgery; PS, Plastic Surgery; GY, Gynecologic surgery; DNT, Dental Surgery.

Table S2. Univariate and multivariable regression analysis model for 90-day mortality in the development cohort

	Univariate analysis		Multivariable analysis		
	OR (95% CI)	*P* value	Estimate	OR (95% CI)	*P* value
Age, years	1·02 (0·98-1·06)	0·400	0·037	1·04 (1·00-1·08)	0·083
Male	1·12 (0·79-1·58)	0·535	0·062	1·06 (0·74-1·53)	0·739
HFRS					
0	1 (reference)			1 (reference)	
1-4	1·44 (0·98-2·11)	0·067	0·436	1·55 (1·04-2·31)	0·033
≥ 5	2·18 (1·34-3·54)	0·002	0·724	2·06 (1·23-3·45)	0·006
Operation Group					
Group 1	1 (reference)			1 (reference)	
Group 2	1·55 (0·65-3·71)	0·326	0·329	1·39 (0·58-3·35)	0·464
Group 3	1·37 (0.55-3.41)	0·505	0·099	1·10 (0·44-2·79)	0·835
Group 4	2·37 (0·93-6·01)	0·070	0·766	2·15 (0·84-5·49)	0·109
Group 5	4·53 (1·96-10·46)	< 0·001	1·427	4·17 (1·79-9·68)	0·001
Group 6	4·72 (1·92-11·58)	0·001	1·437	4·21 (1·70-10·44)	0·002
Group 7	5·76 (2·23-14·88)	< 0·001	1·774	5·90 (2·27-15·33)	< 0·001
Group 8	10·27 (4·09-25·77)	< 0·001	2·243	9·42 (3·70-23·97)	< 0·001

OR, odds ratio; CI, confidence interval; HFRS, hospital frailty risk score.
